# Comprehensive Analyses of Type 1 Diabetes Ketosis- or Ketoacidosis-Related Genes in Activated CD56^+^CD16^+^ NK Cells

**DOI:** 10.3389/fendo.2021.750135

**Published:** 2021-11-25

**Authors:** Ruifeng Shi, Fang Dai, Yong He, Li Sun, Min Xu, Datong Deng, Qiu Zhang

**Affiliations:** Department of Endocrinology, First Affiliated Hospital of Anhui Medical University, Hefei, China

**Keywords:** type 1 diabetes mellitus, ketosis, ketoacidosis, NK cells subset, differentially expressed genes

## Abstract

**Objectives:**

Alterations in natural killer (NK) cells activity cause damage to pancreatic islets in type 1 diabetes mellitus (T1DM). The aim of this study is to identify T1DM ketosis- or ketoacidosis-related genes in activated CD56^+^CD16^+^ NK cells.

**Methods:**

Microarray datasets were downloaded from the Gene Expression Omnibus (GEO) database. Differentially expressed genes (DEGs) were analyzed using the GEO2R tool. Enrichment analyses were performed using Metascape online database and GSEA software. Cell-specific gene co-expression network was built using NetworkAnalyst tools. Cytoscape software was used to identify hub genes and construct co-expressed networks. Target miRNAs were predicted based on the DIANA-micro T, miRDB, and miRWalk online databases.

**Results:**

A total of 70 DEGs were identified between T1DM patients recovered from ketosis or ketoacidosis and healthy control blood samples in GSE44314. Among the DEGs, 10 hub genes were screened out. The mature NK cell-specific gene co-expression network for DEGs in T1DM was built using NetworkAnalyst tools. DEGs between activated CD56^+^CD16^+^ NK cells and CD56^bright^CD16^-^ NK cells were identified from GSE1511. After intersection, 13 overlapping genes between GSE44314 and GSE1511 microarray datasets were screened out, in which 7 hub genes were identified. Additionally, 59 target miRNAs were predicted according to the 7 hub genes. After validating with the exosome miRNA expression profile dataset of GSE97123, seven differentially expressed miRNAs (DEmiRNAs) in plasma-derived exosome were selected. Finally, a mRNA–miRNA network was constructed, which was involved in the T1DM ketosis or ketoacidosis process.

**Conclusion:**

This work identified seven hub genes in activated CD56^+^CD16^+^ NK cells and seven miRNAs in plasma-derived exosome as potential predictors of T1DM ketoacidosis, which provided a novel insight for the pathogenesis at the transcriptome level.

## Introduction

Diabetic ketoacidosis (DKA) is one kind of serious acute hyperglycemic complication of type 1 diabetes mellitus (T1DM) (1). DKA can be a preliminary sign of T1DM and is associated with high morbidity and mortality without optimal treatment. The cost of hospital treatment for patients experiencing DKA exceeds the cost of routine treatment for diabetes (2). The most common causes of DKA include inadequate dose of insulin, infection, intercurrent illness, and drugs (3). For some patients, DKA is an initial manifestation of T1DM (4). Therefore, early identification of DKA-related gene expression is of great significance for prevention.

The development of T1DM is associated with various immune cell populations as it is a multifactorial autoimmune disease (5–8). Natural killer (NK) cells as a crucial component of immune system are also involved in this process. The inhibitory receptors and activating receptors expressed on human NK cells have been demonstrated to play a vital role in pathological situations (9, 10). Additionally, NK cells can secrete cytokine participating in kinds of immune regulation. Recent studies focus on the association between NK cells and autoimmune diabetes. The frequencies and counts of NK cells in peripheral blood were reported to be altered in newly diagnosed T1DM patients (11, 12). The peripheral blood of LADA patients also exhibited a significant decrease in NK cells frequency (13). Therefore, NK cells might serve as a primary immune regulator in T1DM due to their immunoregulatory properties. However, only the activated NK cell subset is abundant with cytokine producers (14).

Human NK cells are divided into different populations based on their cell surface density of CD56 and CD16 (15). The CD56^bright^CD16^-^ NK cells are inactivated and low cytotoxic NK cells, while the CD56^+^CD16^+^ NK cells are activated NK cells in inflamed tissue (16). Inflammation has a broad role in T1DM, which contributes to the induction and amplification of immune assault against islet β cells (17). As activated CD56^+^CD16^+^ NK cells in T1DM have been investigated scarcely, this study was to explore potential genes in activated CD56^+^CD16^+^ NK cells associated with T1DM ketosis or ketoacidosis. Exosomes are important mediators in cell communication. Evidence shows a distinct exosome miRNA signature in T1DM (18). Since a better understanding of exosome miRNA may provide novel insight into DKA, exosome miRNAs related to NK cells in T1DM will also be explored in our study.

## Materials and Methods

### Microarray Data Acquisition and Processing

The gene expression profiles including GSE44314, GSE1511, and GSE97123 were downloaded from the Gene Expression Omnibus (GEO, https://www.ncbi.nlm.nih.gov/geo/). The microarray profile dataset GSE44314, deposited by Shinsuke et al., was conducted on blood samples from five classical type 1A diabetes recovered from ketosis or ketoacidosis and six healthy controls. The gene expression dataset GSE1511, provided by Mandelboim et al., was selected for containing CD56^bright^CD16^-^ and activated CD56^+^CD16^+^ human NK cell subset samples. The miRNA (in plasma-derived exosomes) expression profile GSE97123 included 12 T1DM and 12 control subjects. The differentially expressed genes (DEGs) and differentially expressed miRNAs (DEmiRNAs) were analyzed using online tool GEO2R (https://www.ncbi.nlm.nih.gov/geo/geo2r/). The *p*-value < 0.05 and |log2 (Fold-Change)| ≥ 1 were considered criteria for DEGs. Overlapping genes were identified by the Venn diagram webtool (http://bioinformatics.psb.ugent.be/webtools/Venn/).

### Enrichment Analysis

The Metascape online database (http://metascape.org) was used for Gene Ontology (GO) annotation analysis and Kyoto Encyclopedia of Genes and Genomes (KEGG) pathway enrichment analysis of DEGs (19). The enriched KEGG pathway and GO terms were selected with a criterion of *p* < 0.05. The gene expression information of all T1DM was uploaded to GSEA software to assess the distribution trend of genes, which determine their contribution to the phenotype (20). Cell-specific gene co-expression network was built using NetworkAnalyst tools (21).

### Construction of a PPI Network

A PPI (protein–protein interaction) network was built with Search Tool for the Retrieval of Interacting Genes (STRING) database (http://string-db.org/) (22). The cytoHubba plugin in Cytoscape software (version 3.7.2) was used to select hub genes and construct mRNA–miRNA network.

### Prediction and Validation of Target miRNA

Online miRNA databases DIANA-micro T, miRDB, and miRWalk were used to predict target miRNAs based on hub genes (23–25). In addition, the DEmiRNA in GSE97123 was analyzed. Only the overlapping miRNA that was found in all the above three databases and verified with the GSE97123 dataset was considered as target miRNA.

## Results

### Identification and Enrichment Analysis of Differentially Expressed Genes in T1DM Ketosis or Ketoacidosis

**Figure 1:**
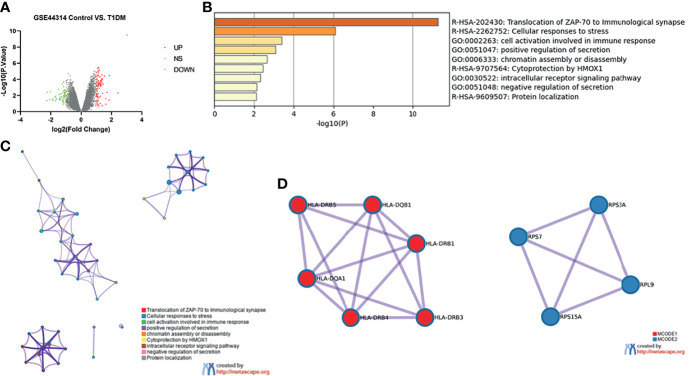
Differentially expressed genes in T1DM ketosis or ketoacidosis. **(A)** Volcano plot for differentially expressing genes between T1DM recovered from ketosis or ketoacidosis and healthy control samples. Red dots represented upregulated genes, while green dots represented downregulated genes. **(B)** Heatmap of Gene Ontology (GO) and Kyoto Encyclopedia of Genes and Genomes (KEGG) enriched clusters colored by *p*-value. **(C)** Network of GO and KEGG enriched terms colored by clusters. **(D)** MCODE algorithm was applied to this network to identify neighborhoods. Each MCODE network was assigned a unique color. GO enrichment analysis was applied to each MCODE network to assign “meanings” to the network component.

The dataset GSE44314 contained five classical T1DM recovered from ketosis or ketoacidosis and six healthy controls. There was no difference in gender (male: 20.0% *vs*. 33.3%, *p* > 0.05) or age (53.8 ± 7.6 *vs*. 47.0 ± 6.4, *p* > 0.05) between the two groups in GSE44314 (**Table S1**). As shown in **Figure 1A**, a total of 70 DEGs were identified, including 32 upregulated genes and 38 downregulated genes. The GO and KEGG enrichment analyses were performed using Metascape online tools. In enriched functional category terms, five enriched GO terms and four KEGG pathways were identified (**Figure 1B**). A network of GO and KEGG enriched terms colored by clusters is shown in **Figure 1C**. MCODE algorithm was applied to this network to identify neighborhoods where proteins were densely connected (**Figure 1D**).

### GSEA Analysis of T1DM Ketosis- or Ketoacidosis-Related Genes

**Figure 2:**
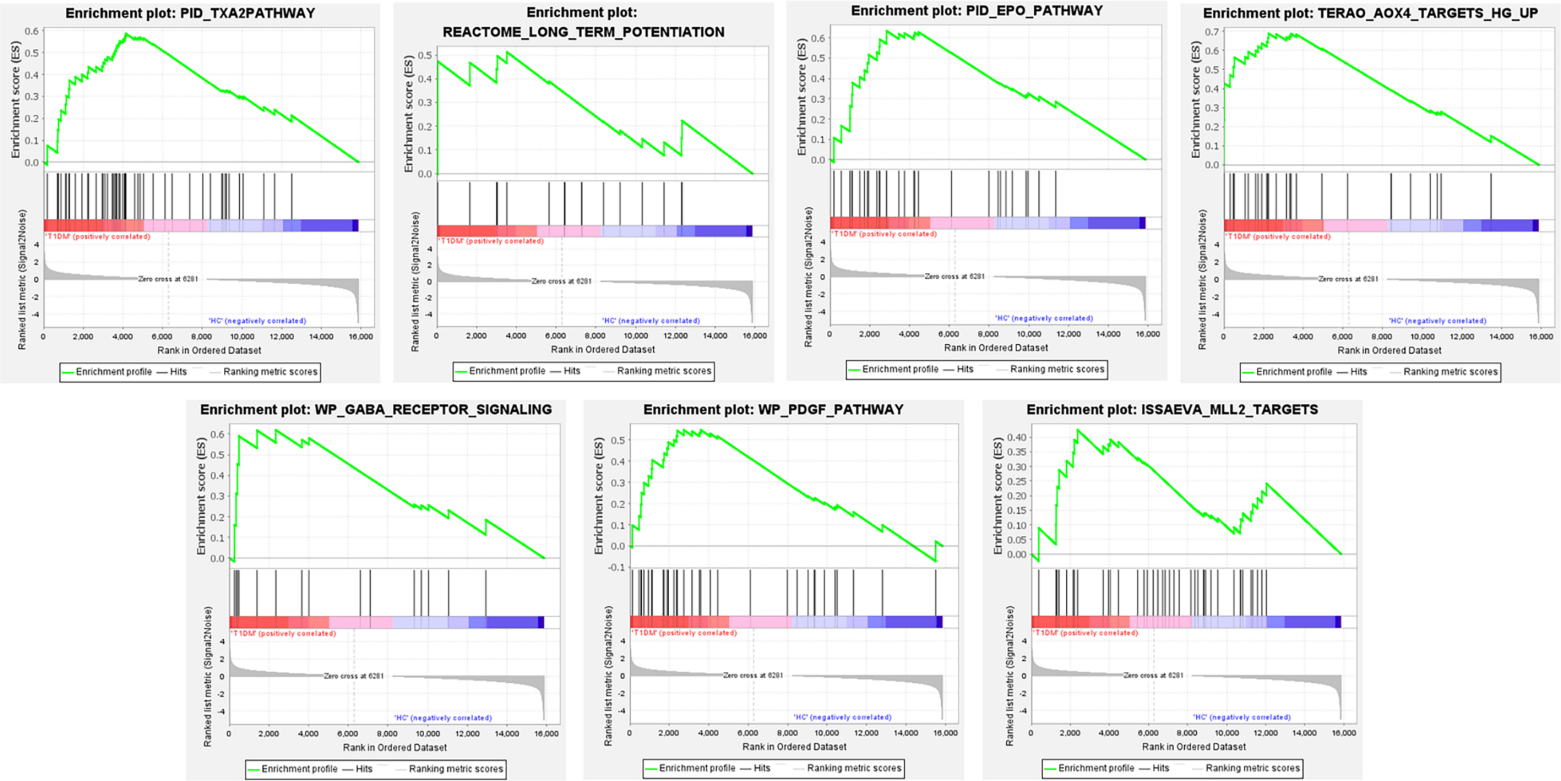
The whole gene expression value of T1DM ketosis or ketoacidosis and control samples analyzed by GSEA software. The top seven gene sets were enriched with criterion of NES>2.5, FDR < 0.25, and *p*-value < 0.01.

The gene in expression profile was analyzed using Molecular Signatures Database at a holistic level *via* GSEA software. A total of 2,565 gene sets were upregulated in the classical T1DM samples compared to healthy controls. A total of 1,046 gene sets were significantly enriched at FDR < 0.25 and 1,194 gene sets were significantly enriched at nominal *p*-value < 0.01. The enrichment results of GSEA analysis showed pathways enriched in the T1DM ketosis or ketoacidosis samples (**Table S2**). As shown in **Figure 2**, the top seven gene sets with NES>2.5 were PID_TXA2PATHWAY, REACTOME_LONG_TERM_POTENTIATION, PID_EPO_PATHWAY, TERAO_AOX4_TARGETS_HG_UP, WP_GABA_RECEPTOR_SIGNALING, WP_PDGF_PATHWAY, and ISSAEVA_MLL2_TARGETS.

### Hub Gene Identification of T1DM Ketosis or Ketoacidosis

**Figure 3:**
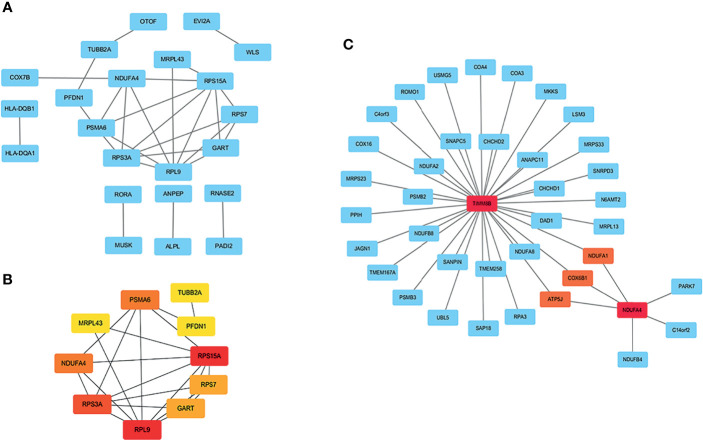
PPI network analysis of DEGs in T1DM ketosis or ketoacidosis. The node represented gene and the edge represented link between genes. **(A)** PPI network of differentially expression genes in T1DM ketosis or ketoacidosis. **(B)** Cytoscape network clustering visualization of hub DEGs in T1DM ketosis or ketoacidosis samples. The node represented gene and the edge represented link between genes. The red/orange/yellow color meant the MMC score was highest/moderate high/mild high. **(C)** The mature NK cell-specific gene co-expression network for DEGs in T1DM ketosis or ketoacidosis.

A PPI network with 63 nodes and 28 edges was obtained using the STRING tools (**Figure S1**). The CytoHubba plugin in Cytoscape software was used to cluster network genes to identify hub genes in network. The network with an interaction score >0.4 was built according to the STRING online database (**Figure 3A**). The edge represented the link between different genes. As shown in **Figures 3B**, a key module with eight upregulated genes (RPS15A, RPS7, RPL9, RPS3A, NDUFA4, MRPL43, PSMA6, and PFDN1) and two downregulated genes (GART and TUBB2A) was identified. It was consistent with **Figure 1D** in which one module was made up of RPS15A, RPS7, RPL9, and RPS3A. In addition, cell-specific gene co-expression network was built using NetworkAnalyst tools. The mature NK cell-specific gene co-expression network for DEGs in T1DM ketosis or ketoacidosis is shown in **Figure 3C**.

### Identification and Enrichment of Differentially Expressed Genes in the Activated CD56^+^CD16^+^ Human NK Cell Subset

**Figure 4:**
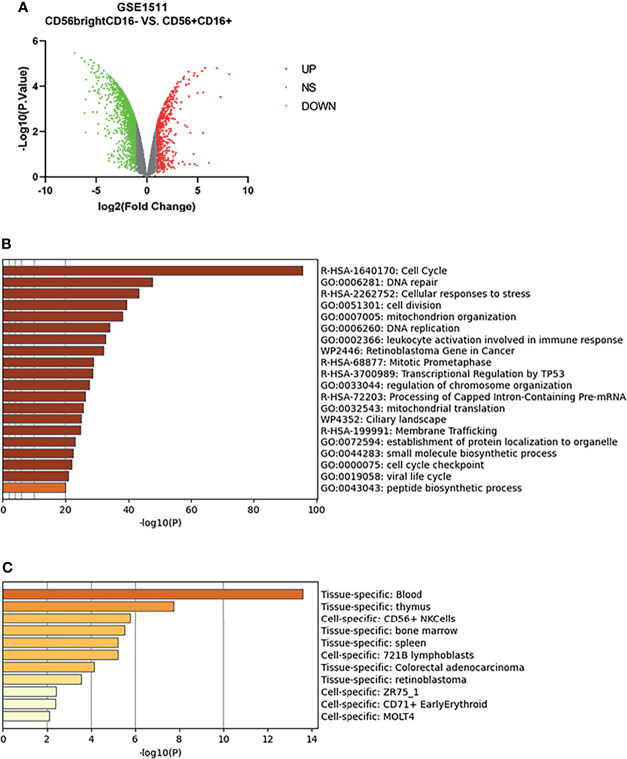
Differentially expressed genes in activated CD56^+^CD16^+^ human NK cell subset samples. **(A)** Volcano plot for differentially expressing genes between CD56^bright^CD16^-^ and activated CD56^+^CD16^+^ human NK cell subset samples. Red dots represented significantly upregulated genes, while green dots represented significantly downregulated genes. **(B)** Heatmap of Gene Ontology (GO) and Kyoto Encyclopedia of Genes and Genomes (KEGG) enriched clusters colored by *p*-value. **(C)** The summary of enrichment analysis in PaGenBase confirmed tissue and cell specific for these genes.

The microarray expression dataset GSE1511 was analyzed to acquire DEGs between CD56^bright^CD16^-^ and activated CD56^+^CD16^+^ human NK cell subsets (**Figure 4A**). A total of 2,671 DEGs were identified including 282 upregulated genes and 2,389 downregulated genes. Enriched GO terms and KEGG pathways were identified using Metascape online tools (**Figure 4B**). Cell cycle, Cellular responses to stress, Retinoblastoma Gene in Cancer, Mitotic Prometaphase, Transcriptional Regulation by TP53, Processing of Capped Intron-Containing Pre-mRNA, and Ciliary landscape were significantly enriched in KEGG pathway. The enriched GO terms included DNA repair, cell division, mitochondrion organization, DNA replication, leukocyte activation involved in immune response, regulation of chromosome organization, mitochondrial translation, establishment of protein localization to organelle, small molecule biosynthetic process, cell cycle checkpoint, viral life cycle, and peptide biosynthetic process (**Table 1**). The summary of enrichment analysis in PaGenBase confirmed tissue and cell specific for these genes (**Figure 4C** and **Table 2**).

**Table 1:** Top clusters with their representative enriched GO terms (one per cluster).

GO	Category	Description	Count	%	Log10(*p*)	Log10(*q*)
GO:0006281	GO Biological Processes	DNA repair	164	7.13	−47.59	−44.28
GO:0051301	GO Biological Processes	Cell division	156	6.79	−39.36	−36.37
GO:0007005	GO Biological Processes	Mitochondrion organization	146	6.35	−38.15	−35.18
GO:0006260	GO Biological Processes	DNA replication	95	4.13	−33.93	−31.14
GO:0002366	GO Biological Processes	Leukocyte activation involved in immune response	162	7.05	−32.62	−29.90
GO:0033044	GO Biological Processes	Regulation of chromosome organization	85	3.70	−27.51	−25.12
GO:0032543	GO Biological Processes	Mitochondrial translation	56	2.44	−25.58	−23.31
GO:0072594	GO Biological Processes	Establishment of protein localization to organelle	124	5.39	−22.99	−20.89
GO:0044283	GO Biological Processes	Small molecule biosynthetic process	138	6.00	−22.38	−20.32
GO:0000075	GO Biological Processes	Cell cycle checkpoint	66	2.87	−21.88	−19.84
GO:0019058	GO Biological Processes	Viral life cycle	87	3.78	−20.77	−18.79
GO:0043043	GO Biological Processes	Peptide biosynthetic process	139	6.05	−19.94	−17.99

“Count” is the number of genes in the given ontology term. “%” is the percentage of all genes that are found in the given ontology term. “Log10(p)” is the p-value in log base 10. “Log10(q)” is the multi-test adjusted p-value in log base 10.

**Table 2:** Summary of enrichment analysis in PaGenBase.

GO	Description	Count	%	Log10(*p*)	Log10(*q*)
PGB:00041	Tissue-specific: blood	58	2.50	−14.00	−11.00
PGB:00016	Tissue-specific: thymus	43	1.90	−7.70	−5.70
PGB:00043	Cell-specific: CD56+ NK Cells	18	0.78	−5.80	−4.00
PGB:00048	Tissue-specific: bone marrow	40	1.70	−5.50	−3.80
PGB:00011	Tissue-specific: spleen	70	3.00	−5.20	−3.50
PGB:00046	Cell-specific: 721B lymphoblasts	21	0.91	−5.20	−3.50
PGB:00101	Tissue-specific: Colorectal adenocarcinoma	12	0.52	−4.10	−2.60
PGB:00060	Tissue-specific: retinoblastoma	19	0.83	−3.50	−2.10
PGB:00111	Cell-specific: ZR75_1	5	0.22	−2.40	−1.10
PGB:00026	Cell-specific: CD71+ Early Erythroid	22	0.96	−2.40	−1.10
PGB:00036	Cell-specific: MOLT4	20	0.87	−2.10	−0.91

### Identification and Cluster Analysis of Overlapping Genes

**Figure 5:**
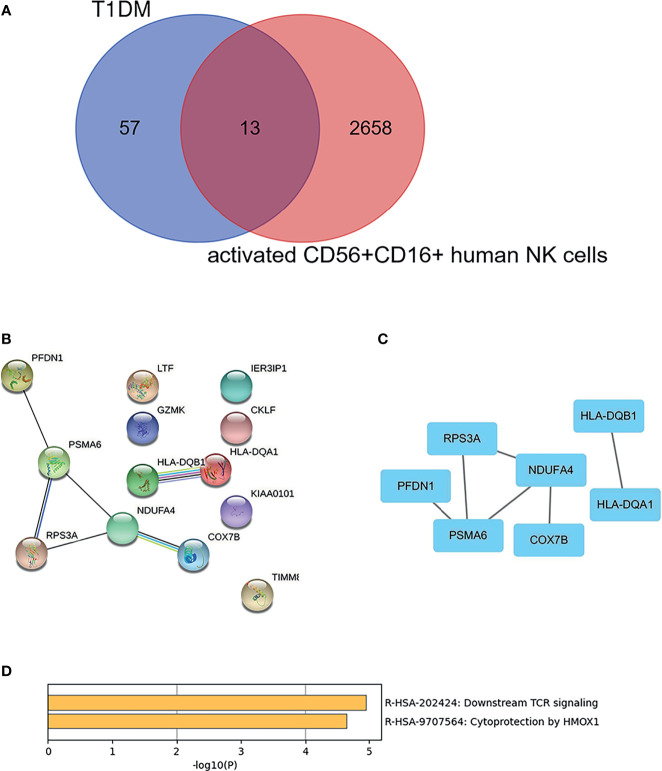
Identification and cluster analysis of overlapping genes. **(A)** Venn diagram of differentially expressed genes between the DEGs in T1DM ketosis or ketoacidosis and DEGs in the activated CD56^+^CD16^+^ human NK cell subset. **(B)** PPI network for the 13 overlapping genes. **(C)** Analysis of hub genes using cytoHubba for the overlapped genes. **(D)** Heatmap of Kyoto Encyclopedia of Genes and Genomes (KEGG) enriched clusters colored by *p*-value.

As shown in **Figure 5A**, there were 13 overlapping genes (COX7B, HLA-DQA1, RPS3A, PFDN1, PSMA6, KIAA0101, HLA-DQB1, GZMK, NDUFA4, CKLF, LTF, TIMM8B, and IER3IP1) between DEGs in T1DM and DEGs in the activated CD56^+^CD16^+^ NK cell subset. Based on the STRING online database, a PPI network with an interaction score >0.4 was constructed (**Figure 5B**). The hub genes (COX7B, HLA-DQA1, RPS3A, PFDN1, PSMA6, HLA-DQB1, and NDUFA4) were identified using Cytoscape software (**Figure 5C**). Enriched KEGG pathways included “Downstream TCR signaling” and “Cytoprotection by HMOX1”, which were identified using Metascape online tools (**Figure 5D**).

### Prediction and Validation of Target miRNA

**Figure 6:**
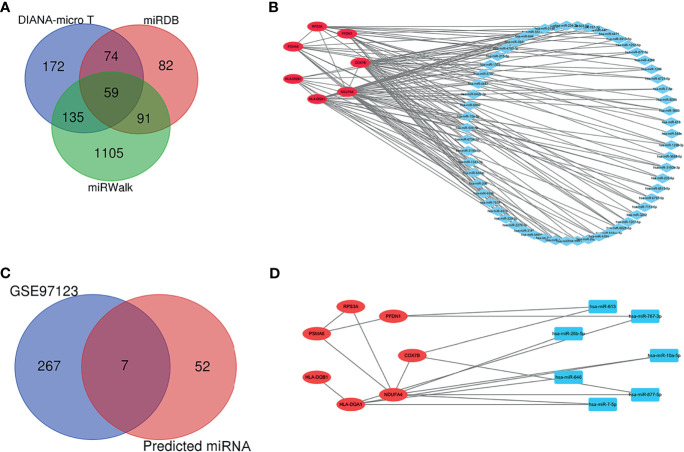
Prediction and validation of target miRNA. **(A)** Venn diagram showing the numbers of predicted miRNAs based on the DIANA-micro T, miRDB, and miRWalk online databases. **(B)** The relationship between 7 hub genes and 59 miRNAs (miRNA–mRNA regulatory network). **(C)** Venn diagram showing the intersection between predicted miRNAs and DEmiRNAs in T1DM patients from the GSE97123 dataset. **(D)** The relationship between seven hub genes and seven validated miRNAs (miRNA–mRNA regulatory network).

The target miRNAs were predicted based on the DIANA-micro T, miRDB, and miRWalk online databases. A total of 1,718 target miRNAs to seven specifically expressed hub genes were obtained. There were 59 miRNAs after intersection (**Figure 6A**). The miRNA–mRNA regulatory network was constructed using CytoHubba (**Figure 6B**). DEmiRNAs in T1DM patients were acquired by analyzing the GSE97123 dataset, in which all miRNAs were obtained from plasma-derived exosome. There was no difference in gender (male: 50.0% *vs*. 50.0%, *p* > 0.05) or age (41.3 ± 2.9 *vs*. 46.4 ± 4.0, *p* > 0.05) between the two groups in GSE97123 (**Table S3**). After intersection with the above 59 predicted miRNAs, 7 miRNAs were screened out (**Figure 6C**). A co-expressed network on T1DM ketosis or ketoacidosis was built based on the predicted miRNA–RNA pairs (**Figure 6D**).

## Discussion

Our study analyzed DEGs between healthy controls and T1DM who recovered from ketosis or ketoacidosis, as well as the DEGs between activated CD56^+^CD16^+^ NK cells and CD56^bright^CD16^-^ NK cells. In order to explore the correlation between T1DM ketone prone and activated CD56^+^CD16^+^ NK cells, the intersection of DEGs in the two data series was performed and 13 overlapped genes were screened out. Moreover, seven hub genes were picked out by PPI analysis. The seven hub genes in activated CD56^+^CD16^+^ NK cells might play a critical role in type 1A diabetes ketosis or ketoacidosis. Based on predicted miRNAs for the seven hub genes and miRNAs in the GSE97123 dataset, seven miRNAs in plasma-derived exosomes were picked out, which might become detectable biomarkers in plasma.

Diabetes increases ketone production and decreases ketone clearance (26). Genetic factors play a dominant role in the progress. The association between genes and the risk for DKA is still unclarified (27). The type 1A diabetes in GSE4431 have been recovered from ketosis or ketoacidosis and treated with insulin intensively for at least 1 month. Therefore, samples from T1DM were free of metabolic derangements and represented the genetic difference for ketosis or ketoacidosis prone. A total of 70 DEGs were identified in our study. Additionally, most of them were clustered according to mature NK cell-specific gene co-expression network. This was in line with a previous study, which reported that NK cells participated in the process of T1DM (28).

Type 1A diabetes is mediated by islet-associated autoimmunity, in which immune cells play a critical role, while the mechanism is still unclarified (17). T cells are proved to infiltrate pancreatic islets, resulting in β cell destruction. However, NK cells are the first type of cells to infiltrate islets without the need for previous activation (28). NK cells have been demonstrated to be involved in several steps of the immune-mediated attack (29). A more recent study shows that NK cell subsets are associated with the partial remission of T1DM (30). Based on CD56 and CD16 expression, human NK cells can be purified into different subpopulations, which have unique repertoires of the chemokine receptor (14, 15, 31). CD56^+^CD16^+^ NK cells can be activated by cytokine IL-21 in combination with IL-15 (32). Moreover, activated CD56^+^CD16^+^ NK cells have been demonstrated to be highly cytotoxic and strikingly distinct from other subsets (16). However, the study on activated CD56^+^CD16^+^ NK cells in T1DM ketone or ketoacidosis is rare.

In our study, DEGs analysis for T1DM with a history of ketone or ketoacidosis and activated CD56^+^CD16^+^ NK cells were combined to identify potential predicted genes for T1DM ketoacidosis. After PPI analysis, seven key genes were identified from 13 overlapping genes, which formed two modules. One module was formed with HLA-DAQ1 and HLA-DBQ1. It is well known that human leukocyte antigen (HLA) class II gene alleles at the DQA1 and DQB1 loci are the major genetic determinants for T1DM (27). According to the GeneCards database (https://www.genecards.org/), five other genes in the other module are involved in cell metabolism. Cytochrome C Oxidase Subunit 7B (COX7B) is the last enzyme in the mitochondrial electron transport chain that drives oxidative phosphorylation. Mitochondrial Complex Associated (NDUFA4) codes protein that has NADH dehydrogenase activity and oxidoreductase activity. Ribosomal Protein S3A (RPS3A) is a protein coding gene. Among its related pathways are metabolism of proteins and HIV Life Cycle. Prefoldin Subunit 1 (PFDN1)-related pathways are metabolism of proteins and cooperation of Prefoldin and TriC/CCT in actin and tubulin folding. Proteasome 20S Subunit Alpha 6 (PSMA6) is a protein coding gene. Among its related pathways are Cellular Senescence (REACTOME) and RET signaling.

The result of enrichment analysis for the seven hub genes included downstream TCR signaling and cytoprotection by HMOX1. T cell receptors (TCRs) play a vital role in T-cell function and immunological synapse formation. TCR activation promotes a number of signaling cascades that determine cell fate through regulating cytokine production, cell survival, proliferation, and differentiation. TCR signaling events drive the progression of T1DM by affecting T-cell development (33). Sequencing of TCR gene in pancreatic islets of T1DM donors reveals repeat clonal expansion and supports the existence of public TCRs shared among T1DM (34–37). Heme Oxygenase 1 (HOMX1) is an essential enzyme in heme catabolism and cleaves heme to form biliverdin, which is subsequently converted to bilirubin by biliverdin reductase, and carbon monoxide, a putative neurotransmitter. Heme oxygenase 1 (HO-1), also named heme oxygenase-1 (HO-1), expression upregulation in high glucose plus oxidized LDL-treated primary peritoneal macrophages from wild-type mice and Nrf2/HO-1 can be a therapeutic target for diabetic nephropathy (38, 39). It is consistent with our enrichment result that downstream TCR signaling and cytoprotection by HMOX1 may be involved in T1DM with ketosis or ketoacidosis. Therefore, the seven key genes in activated CD56^+^CD16^+^ NK cells might play a vital role in T1DM ketosis or ketoacidosis.

Exosome miRNA profiling in different body fluids have been reported to have potential in the diagnosis of disease (40). Exosomes, as small single-stranded non-coding RNAs, have been demonstrated to take part in islet autoimmunity (41). Emerging studies focus on the relationship between exosomes and T1DM development (42). Our study predicted target miRNAs according to seven hub genes. Moreover, the predicted miRNAs were verified with the data from GSE97123, which were derived from plasma exosome. Therefore, the predicted miRNAs in our study could be accurate and detectable predictors for early ketoacidosis. The role of miRNA as a key regulator of mRNA has been well established (43). Accordingly, a co-expressed network of mRNAs–miRNAs was constructed in our study. Nevertheless, our results were based on bioinformatics analysis, which was the limitation of this study. Future research is needed to explore the clinical application value of these potential biomarkers.

In summary, seven hub genes were identified from two datasets. In addition, seven target miRNAs were predicted and validated using GEO expression profiling data. Finally, a miRNA–mRNA network was constructed. Our study provided a reliable comprehensive analysis on the DEGs profile in activated CD56^+^CD16^+^ NK cells for T1DM ketoacidosis, which provided a novel insight for the pathogenesis at transcriptome.

## Data Availability Statement

The datasets presented in this study can be found in online repositories. The names of the repository/repositories and accession number(s) can be found at: https://www.ncbi.nlm.nih.gov/geo/, GSE44314; https://www.ncbi.nlm.nih.gov/geo/, GSE1511; https://www.ncbi.nlm.nih.gov/geo/, GSE97123.

## Author Contributions

RS was involved in the overall study, designed the analysis plan, analyzed the data, and wrote the manuscript. FD, YH, LS, and MX collected the data and analyzed the data. DD and QZ contributed to the discussion and reviewed the manuscript. All authors contributed to the article and approved the submitted version.

## Funding

This study was supported by the National Natural Science Foundation of China (No. 82100845) and the Cultivation Plan of First Affiliated Hospital of Anhui Medical University for National Natural Science Foundation of China Youth Science Foundation in 2021 (2021kj03).

## Conflict of Interest

The authors declare that the research was conducted in the absence of any commercial or financial relationships that could be construed as a potential conflict of interest.

## Publisher’s Note

All claims expressed in this article are solely those of the authors and do not necessarily represent those of their affiliated organizations, or those of the publisher, the editors and the reviewers. Any product that may be evaluated in this article, or claim that may be made by its manufacturer, is not guaranteed or endorsed by the publisher.

## Supplementary Material

Supplementary Figure 1 A PPI network for T1DM ketosis or ketoacidosis obtained using the STRING tools.

Supplementary Table 1 The Characteristic of 5 classical T1DM recovered from ketosis or ketoacidosis and 6 healthy controls samples in GSE44314.

Supplementary Table 2 The enrichment results of GSEA analysis for pathways enriched in the T1DM ketosis or ketoacidosis samples.

Supplementary Table 3 The Characteristic of 12 T1DM and 12 healthy controls samples in GSE97123.
