# Supplementary figures and images for: Comprehensive Analyses of Type 1 Diabetes Ketosis- or Ketoacidosis-Related Genes in Activated CD56+CD16+ NK Cells
Source: Front Endocrinol (Lausanne). 2021 Nov 25;12:750135. doi: 10.3389/fendo.2021.750135 (PMC8656236; doi:10.3389/fendo.2021.750135)

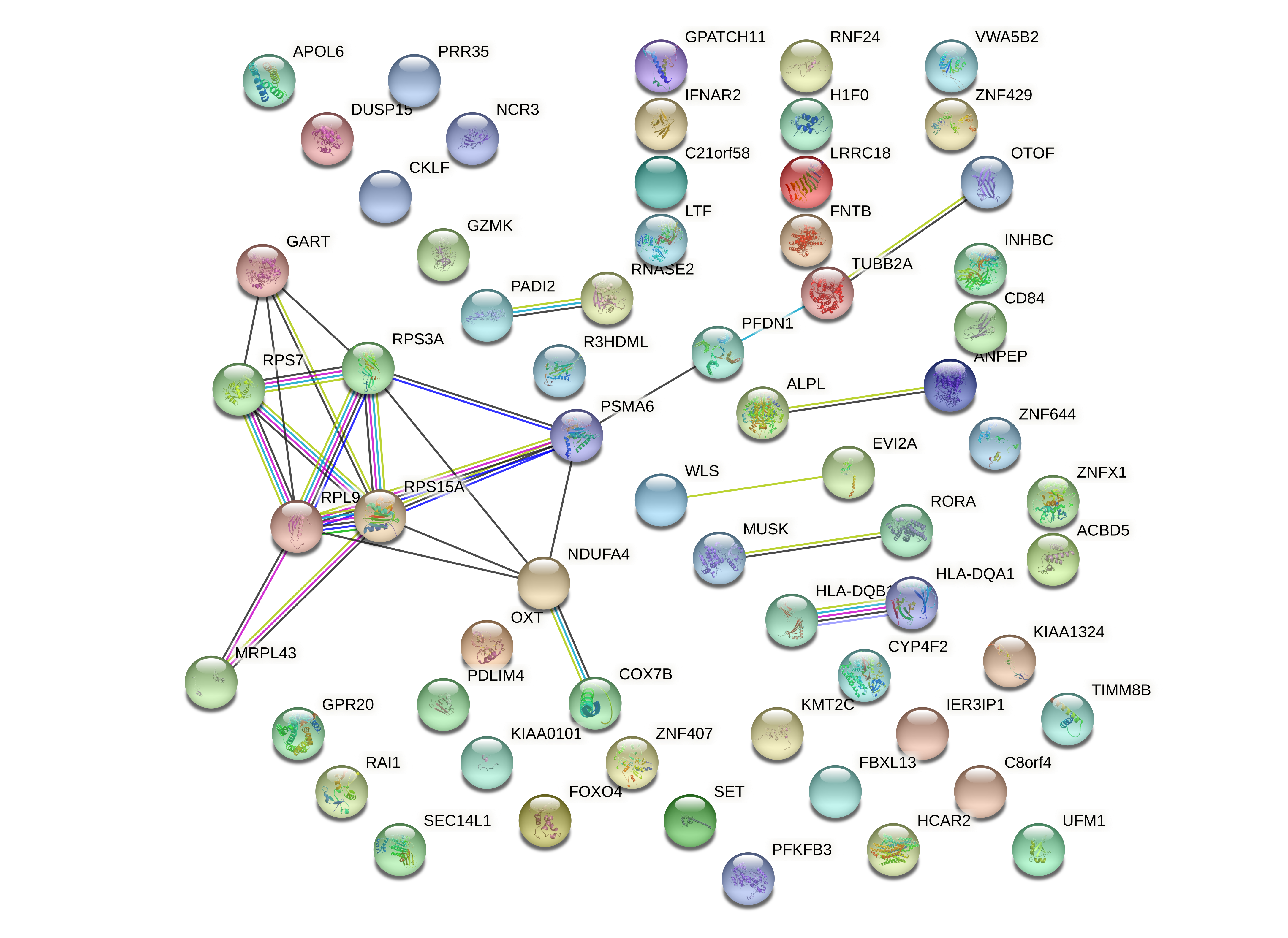

Supplement: Supplementary Figure 1 — A PPI network for T1DM ketosis or ketoacidosis obtained using the STRING tools. [file Image_1.png]
