# Supplementary material for: Comprehensive Analyses of Type 1 Diabetes Ketosis- or Ketoacidosis-Related Genes in Activated CD56+CD16+ NK Cells
Source: Front Endocrinol (Lausanne). 2021 Nov 25;12:750135. doi: 10.3389/fendo.2021.750135 (PMC8656236; doi:10.3389/fendo.2021.750135)
Supplement: Supplementary Table 1 — The Characteristic of 5 classical T1DM recovered from ketosis or ketoacidosis and 6 healthy controls samples in GSE44314. [file Table_1.docx]

Table S1. The Characteristic of 5 classical T1DM recovered from ketosis or ketoacidosis and 6 healthy controls samples in GSE44314.

| Accession | Title | Tissue | Gender | Age(years) | Diabetes type |
| --- | --- | --- | --- | --- | --- |
| GSM1082969 | Classical 1A_1 (2011) | whole blood | female | 67 | classical type 1A |
| GSM1082970 | Classical 1A_2 (2011) | whole blood | female | 40 | classical type 1A |
| GSM1082971 | Classical 1A_3 (2011) | whole blood | male | 49 | classical type 1A |
| GSM1082972 | Classical 1A_4 (2011) | whole blood | female | 37 | classical type 1A |
| GSM1082973 | Classical 1A_5 (2011) | whole blood | female | 76 | classical type 1A |
| GSM1082974 | Healthy control_1 | whole blood | male | 67 | none |
| GSM1082975 | Healthy control_2 | whole blood | female | 67 | none |
| GSM1082976 | Healthy control_3 | whole blood | male | 39 | none |
| GSM1082977 | Healthy control_4 | whole blood | female | 39 | none |
| GSM1082978 | Healthy control_5 | whole blood | female | 35 | none |
| GSM1082979 | Healthy control_6 | whole blood | female | 35 | none |
