# Supplementary material for: Comprehensive Analyses of Type 1 Diabetes Ketosis- or Ketoacidosis-Related Genes in Activated CD56+CD16+ NK Cells
Source: Front Endocrinol (Lausanne). 2021 Nov 25;12:750135. doi: 10.3389/fendo.2021.750135 (PMC8656236; doi:10.3389/fendo.2021.750135)
Supplement: Supplementary Table 3 — The Characteristic of 12 T1DM and 12 healthy controls samples in GSE97123. [file Table_3.docx]

Table S3. The Characteristic of 5 classical T1DM recovered from ketosis or ketoacidosis and 6 healthy controls samples in GSE97123

| Accession | Title | Tissue | Tissue compartment | Diagnosis | Age(years) | Sex |
| --- | --- | --- | --- | --- | --- | --- |
| GSM2551959 | Control 1 | Plasma | exosome | Control | 33 | Male |
| GSM2551960 | Control 2 | Plasma | exosome | Control | 36 | Male |
| GSM2551961 | Control 3 | Plasma | exosome | Control | 57 | Female |
| GSM2551962 | Control 4 | Plasma | exosome | Control | 39 | Female |
| GSM2551963 | Control 5 | Plasma | exosome | Control | 33 | Female |
| GSM2551964 | Control 6 | Plasma | exosome | Control | 31 | Female |
| GSM2551965 | Control 7 | Plasma | exosome | Control | 34 | Male |
| GSM2551966 | Control 8 | Plasma | exosome | Control | 37 | Male |
| GSM2551967 | Control 9 | Plasma | exosome | Control | 36 | Female |
| GSM2551968 | Control 10 | Plasma | exosome | Control | 57 | Female |
| GSM2551969 | Control 11 | Plasma | exosome | Control | 46 | Male |
| GSM2551970 | Control 12 | Plasma | exosome | Control | 56 | Male |
| GSM2551971 | T1D 13 | Plasma | exosome | Type 1 Diabetes | 35 | Male |
| GSM2551972 | T1D 14 | Plasma | exosome | Type 1 Diabetes | 33 | Male |
| GSM2551973 | T1D 15 | Plasma | exosome | Type 1 Diabetes | 57 | Female |
| GSM2551974 | T1D 16 | Plasma | exosome | Type 1 Diabetes | 33 | Female |
| GSM2551975 | T1D 17 | Plasma | exosome | Type 1 Diabetes | 39 | Female |
| GSM2551976 | T1D 18 | Plasma | exosome | Type 1 Diabetes | 62 | Male |
| GSM2551977 | T1D 19 | Plasma | exosome | Type 1 Diabetes | 41 | Female |
| GSM2551978 | T1D 20 | Plasma | exosome | Type 1 Diabetes | 44 | Male |
| GSM2551979 | T1D 21 | Plasma | exosome | Type 1 Diabetes | 26 | Female |
| GSM2551980 | T1D 22 | Plasma | exosome | Type 1 Diabetes | 64 | Female |
| GSM2551981 | T1D 23 | Plasma | exosome | Type 1 Diabetes | 62 | Male |
| GSM2551982 | T1D 24 | Plasma | exosome | Type 1 Diabetes | 61 | Male |
